# Inflammatory Regulation Effect and Action Mechanism of Anti-Inflammatory Effective Parts of Housefly (*Musca domestica*) Larvae on Atherosclerosis

**DOI:** 10.1155/2013/340267

**Published:** 2013-02-28

**Authors:** Fu Jiang Chu, Xiao Bao Jin, Yin Ye Xu, Yan Ma, Xiao Bo Li, Xue Mei Lu, Wen Bin Liu, Jia Yong Zhu

**Affiliations:** ^1^Guangdong Provincial Key Laboratory of Pharmaceutical Bioactive Substances, Guangdong Pharmaceutical University, Guangzhou Higher Education Mega Center, 280 Wai Huan Dong Road, Guangzhou 510006, China; ^2^School of Basic Courses, Guangdong Pharmaceutical University, Guangzhou Higher Education Mega Center, 280 Wai Huan Dong Road, Guangzhou 510006, China

## Abstract

The protein-enriched extracts of housefly larvae were segregated by gel-filtration chromatography (GFC) and then anti-inflammatory activity screening in RAW264.7 (induced by LPS) was carried out. After acquire the anti-inflammatory effective parts, its anti-atherosclerotic properties *in vivo* were then evaluated. Results showed that the anti-inflammatory effective parts of housefly larvae were low-molecular-weight parts. After treated with the effective parts oral gavaged for 4 weeks, the atherosclerotic lesions of the mouse were significantly decreased. The inflammatory and lipid parameters were also reduced (except HDL which was increased). Western blot analysis demonstrated that the effective parts exerted potent inhibitory effect on expression of p65 in nucleus and cytoplasm. The results of immunofluorescence microscopy analysis also showed that the expressions of p65 both in cytoplasm and nucleus were significantly reduced. The hypothesis that the anti-inflammatory effective parts of housefly larvae possessed anti-atherosclerosis activity in mouse and the possible mechanism could be associated with the inhibition of expression and nuclear transfer of NF-**κ**B p65 could be derived.

## 1. Introduction

Insects and insect derivatives have been widely used in folk medicine across the world since ancient times [1]. The use of insects is particularly common in China, and in many other countries including Brazil, Mexico, India, Africa, and South Korea [2]. At present, there are approximately 300 medicinal insects distributed in 70 genera, 63 families, and 14 orders. An estimated 1700 traditional Chinese medicine prescriptions include medicinal insects or insect-derived crude drugs [3]. The housefly (*Musca domestica*) belongs to the order of Diptera. The housefly larvae have been used clinically to cure malnutritional stagnation, decubital necrosis, osteomyelitis, ecthyma, and lip boil which was described by Li Shizhen (1518–1593 AD) in the pharmaceutical text of *Compendium of Materia Medica *[4]. Recently, effects of antioxidant [5], antibacterial, and *in vitro *antitumor properties of the protein extracts of housefly larvae have been reported [6, 7]. Additionally, the oil of housefly larva could be used as a natural ointment to heal burn wound [8]. In our earlier studies, we studied whether the protein-enriched fraction/extracts of housefly larva could potently inhibit multiple proinflammatory responses in atherosclerotic lesions. The results showed that the concentrations of TC, TG, and LDL were lower in the extracts treatment group than in the negative control animal which was treated with cholesterol-enriched diet and LPS (intraperitoneal injection). Also the expressions of TNF-*α*, IL-1*α*, and MCP-1 were also decreased after treated with the extracts *in vitro* [9]. However, the composition of the protein-enriched extracts of housefly larva is very complex and also the specific antiatherosclerotic mechanism is not clear. So the objective of this study was to screen the anti-inflammatory effective parts of housefly larvae and to investigate inflammatory regulation effect and action mechanism of those parts on atherosclerosis. In the present study, the anti-inflammatory effective parts of housefly larvae were obtained by gel-filtration chromatography and (macrophages induced by LPS) anti-inflammatory activity screening* in vitro*. Then the antiatherosclerotic effects in mouse, changes of inflammation-related factors, and expression and nuclear transfer of NF-*κ*B p65 in macrophages were also examined.

## 2. Materials and Methods

### 2.1. Preparation of Extracts of Housefly Larvae

After the protein-enriched fraction/extracts of housefly larva were harvested [9], the extracts were redissolved in deionized-distilled water (DDW), and the supernatant was collected by centrifuge at 3000 r/min, 4°C for 10 minutes, and concentrated by solid polyethylene glycol. The gel filtration material used in the experiment was Sephadex G-75. Sephadex G-75 exposed in 0.05 mol/L Tris buffer (pH 7.5) was incubated in boiling water for 30 min to the column (2.5 cm × 100 cm). The column was equilibrated with 0.05 mol/L Tris buffer (pH 7.5) at a constant flow rate of 0.25 mL/min. The concentrated extracts were applied to the column and eluted with the same buffer. 1.5 mL fractions were collected at a flow rate of 0.25 mL/min. Protein profile was monitored by measuring the absorbance at 280 nm. The fractions were combined at the peaks of elution curves and dialyzed overnight against 0.02 mol/L Tris buffer (pH 8.5), and then the vacuum freeze-drying was used to remove the solvents for the next step.

### 2.2. Screening of Housefly Larvae Extracts for Anti-Inflammatory Activity

#### 2.2.1. Cell Culture

The mouse macrophage cell line, RAW264.7, was purchased from Cell Bank, Center of Experimental Animals, Sun Yat-Sen University. Guangzhou, China. RAW264.7 cells grown in Dulbecco's modified Eagle's medium (DMEM) were supplemented with 10% (v/v) endotoxin-free fetal calf serum, 2 mmol glutamine/L, and 100 U/mL penicillin/streptomycin at 37°C in atmosphere of 10%  CO_2_ and 95% relative humidity.

#### 2.2.2. Cell Activation and Treatment

1 × 10^5^ RAW264.7 cells were plated in 24-well plates, incubated for 24 h and pretreated with the indicated concentrations of the extracts of housefly larvae (40 *μ*g/mL), or a vehicle for another 2 h, then challenged with LPS (1 *μ*g/mL) for an additional 18 h.

#### 2.2.3. Measurement of TNF-*α* and IL-6

The culture supernatants were collected. Supernatant levels of tumor necrosis factor alpha (TNF-*α*) and interleukin 6 (IL-6) in cultured RAW cells were measured by ELISA according to the manufacturer's instructions. The requested solutions were provided with the ELISA compact kits and additional toolkits. Within 30 minutes, optical density of each well was determined by a microplate reader setting at 450 nm, and the correction wavelength setting at 570 nm. 

#### 2.2.4. Evaluation of Cell Morphology

At the indicated times after treatment of different extracts of housefly larvae, the cell-culture medium were washed with PBS, and changes in cell morphology were examined using a microscope with computer imaging system analysis. Cells were counted in four fields of vision, and the total number was determined. Then the change of cellular morphology was observed, and the number of pseudopodia (foot like projections) of each cell was counted.

### 2.3. Evaluation of Antiatherosclerosis Properties

#### 2.3.1. Animals

Healthy female C57BL/6 mice at age of 6 weeks old were purchased from Medical Laboratory Animal Center (Guangzhou, Guangdong, China). All animals received humane care and the present study was carried out in accordance with the guidelines for the humane treatment of animals set by the Association of Laboratory Animal center at Guangdong Pharmaceutical University, Guangzhou, China. All mice were housed in individual cages respectively and kept for at least 1 week with commercial solid diet under controlled conditions (25 ± 2°C, 12 h light/dark cycle, 55 ± 5% humidity) with free access to food and water before treatment.

#### 2.3.2. Treatment

After acclimatization, mice were divided into 2 groups: natural control group (*n* = 6) and model group (*n* = 12). Mice in natural control group were treated with standard diet, whereas mice in model group were treated with LPS (2 mg/kg, i.v.) three times a week, and atherogenic solution (Fat-soluble solution was composed of 20% cholesterol and 80% peanut oil, and water-soluble solution was composed of 10% glucose, 10% sodium cholate, and 80% distilled water, gavage) to induce atherosclerosis. All animals had free access to food and water. After the model was established, all mice in model group were divided into anti-inflammatory effective parts of housefly larvae treated group (200 mg/kg, gavage; *n* = 6) and negative control group (distilled water equivalent to the same dose, gavage; *n* = 6) and received the treatment once a day for 4 weeks.

#### 2.3.3. Measurement of Blood Biochemical Variables

After 4 weeks, all mice were killed, and their serum was analyzed for elevation of total cholesterol (TC), triglyceride (TG), low density lipoprotein cholesterol (LDL-C), and high density lipoprotein cholesterol (HDL-C). Serum lipids were measured using commercially available kits. Total cholesterol, HDL-C, and triglycerides were measured by standardized automated methods, and LDL-C was calculated by the Friedewald equation: LDL-C = TC − HDL-C − TG/5. The levels of L-6 and TNF-*α* were also determined (refer to Section 2.2.3).

#### 2.3.4. Histology and Morphometric Analysis

A section of the thoracic aorta and myocardial tissues were fixed in 100 mL/L buffered formalin for 24 hours. The tissue was embedded in paraffin, and a 4 *μ*m section was examined through hematoxylin and eosin (HE) staining. Then the histological slides were imaged under bright field using a digital color CCD camera.

### 2.4. Analysis of Expression and Nuclear Transfer of Nuclear Factor *κ*B (NF-*κ*B) p65

#### 2.4.1. Cell Culture and Treatment

The RAW264.7 cells were plated in 24-well plates, incubated for 24 h and pretreated with the extracts of housefly larvae (40 *μ*g/mL), then challenged with LPS (1 mg/mL) for additional 18 h.

#### 2.4.2. Western Blot Analysis of Cytoplasmic and Nuclear Protein Extracts from RAW264.7 Cells

Cytoplasmic and nuclear extracts were prepared from cells using the NE-PER Nuclear and Cytoplasmic Extraction Reagents kit. Protein contents were measured taking bovine serum albumin as a criterion. Protein concentration was determined by a commercial protein assay reagent. Proteins of cytoplasmic and nuclear extracts were separated by sodium dodecyl sulfate polyacrylamide gel electrophoresis (SDS-PAGE). All gels were electrically transferred to PVDF membranes before being blocked for 1 h at RT in 0.01% TBS/Tween containing 3% nonfat milk powder (TBS/Tween with nonfat milk). Membranes were incubated with primary antibodies overnight at 4°C in TBS/Tween with nonfat milk and then washed three times for 5 min in TBS/Tween. Visualization of proteins was performed via the addition of a secondary antibody conjugated to horseradish peroxidase (HRP), which was incubated for 1 h at RT in TBS/Tween with nonfat milk. Membranes were washed three times for 10 min in TBS/Tween. Then protein-antibody complexes were visualized using Vectastain ABC and TMB (3,3′,5,5′-tetramethylbenzidine) substrate kits. Protein bands and NF-*κ*B p65 (65 kDa) and *β*-actin (42 KD) scanning and quantification of signal intensities were performed using a Bio-Rad Gel Doc XR densitometer with Quantity One software. Antibodies were used in the following dilutions: rabbit anti-mouse NF-*κ*B p65 (dilution 1 : 500), anti-mouse *β*-actin (dilution 1 : 500). All secondary antibodies were used at the following dilutions: goat anti-rabbit IgG, 1 : 1000, goat anti-mouse IgG, 1 : 1000.

#### 2.4.3. Immunofluorescence-Microscopy Analysis of Nuclear Transfer NF-*κ*B p65

Briefly, cells were fixed in 3.7% (wt/vol) formaldehyde in PBS for 10 min and permeated with acetone-methanol (1 : 1, vol/vol) at −20°C for 15 min. Following a 30 min blocking in PBS with 3% (wt/vol) bovine, serum albumin samples were exposed to rabbit polyclonal anti-p65 (PE-conjugated). Antibodies were used at a 1 : 100 (vol/vol) diluted in blocking solution for 1 h at 20°C, washed with PBS, and counterstained with 4′,6-diamidino-2-phenylindole (DAPI) (5 min) for the identification of nuclei. Coverslips were finally mounted on mounting medium and fluorescent images were taken under the Laser Confocal Microscope.

### 2.5. Statistical Analysis

All data were expressed as the mean ±  standard error of the mean (SEM). Each measurement was performed at least in triplicate. The Student's *t*-test was applied to compare means of data of two independent samples. For multiple comparisons, one-way analysis of variance (ANOVA) was performed followed by the Dunnett's T 3 (i.e., equal variance was assumed) or Duncan's *t* tests (i.e., equal variance was not assumed) where appropriate. Differences were considered statistically significant when *P* < 0.05. All statistical analysis was performed with SPSS statistical software (version 13.0 for Windows).

## 3. Results

### 3.1. Chromatographic Behavior of Housefly Larvae Extracts

**Figure 1:**
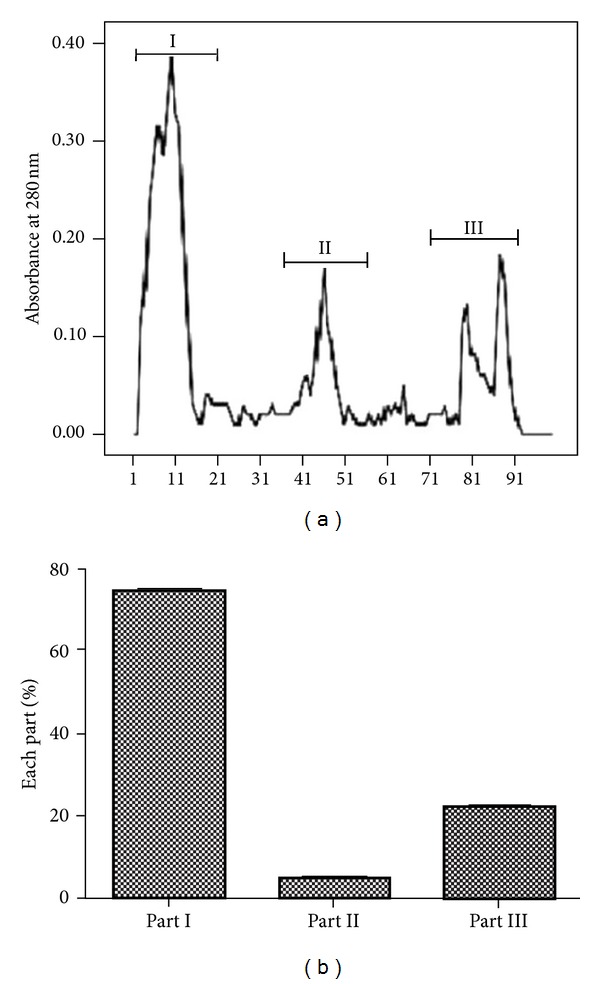
Gel filtration profile (Sephadex G-75) of housefly larvae extracts and percentage of each part.

Gel chromatography with Sephadex G-75 (separated molecules with molecular weights from 3,000 to 70,000) was used for segregation and purification of the extracts of housefly larvae. According to the basic principles of chromatography, the small molecules entering into the interior of the gel should be eluted out of the system. Molecular weight distributions and elution curve were shown in Figure 1. The active fractions of major peaks were pooled, concentrated, and loaded according to the molecular weight (MW) which were recovered in three main fractions of the void volume (part I, high MW; part II, middle MW; part III, low MW). The percentage of each part was also shown.

### 3.2. Anti-Inflammation Activity Screening of Gel-Filtration Chromatography Fractions of Housefly Larvae Extracts

The concentrations of TNF-*α* and IL-6 in the RAW264.7 cell supernatants were measured by ELISA. RAW264.7 cells treated with LPS alone resulted in significant increases in cytokine production compared to the control group. Compared with the LPS-treated group, part I and part II treated group, the levels of TNF-*α* and IL-6 were both significantly decreased in part III treated group (*P* < 0.01 for all). Although the cytokines in part II treated group also decreased compared with that in LPS-treated group, the decrease was not as significant as that of part III treated group (Table 1).

**Figure 2:**
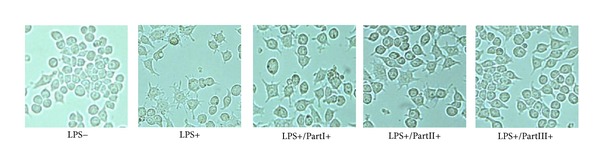
Morphological change in macrophage RAW264.7 cells after treated with/without different parts of extracts of housefly larvae (×400).

The cell morphology of the macrophage RAW264.7 cells treated with different fractions of housefly larvae extracts in the presence or absence of LPS was presented in Figure 2. In the non-LPS-stimulated cells, the cell morphology generally showed a round form, whereas in the LPS-activated RAW264.7 cells, the cell morphology changed into an irregular form with pseudopodia developing rapidly. The cotreatment of LPS with part III inhibited the cell spread and formation of pseudopodia by suppressing cell differentiation. But it was not evident in the other two groups.

The above observations indicate that compared with other fractions (part I and II), part III had more significant anti-inflammatory activity in LPS-stimulated RAW264.7 cells. In general, these results support the hypothesis that part III of housefly larvae extracts was the anti-inflammatory effective parts.

### 3.3. Antiatherosclerosis Properties of the Anti-Inflammatory Effective Parts of Housefly Larvae in Mouse

**Figure 3:**
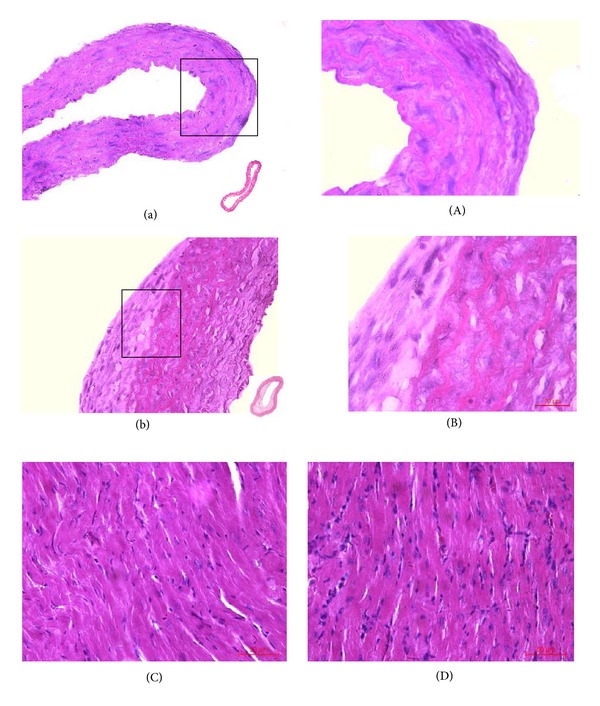
Histology and morphology of thoracic aorta and myocardial tissues treated with LPS (i.v.) and atherogenic solution (gavage) for 8 weeks. (a) Thoracic aorta in normal control group (×200). (b) Thoracic aorta in model group (×200). (A) Thoracic aorta in normal control group (×400). (B) Thoracic aorta in model group (×400). (C) Myocardial tissues in normal control group (×400). (D) Myocardial tissues in model group (×400).

**Figure 4:**
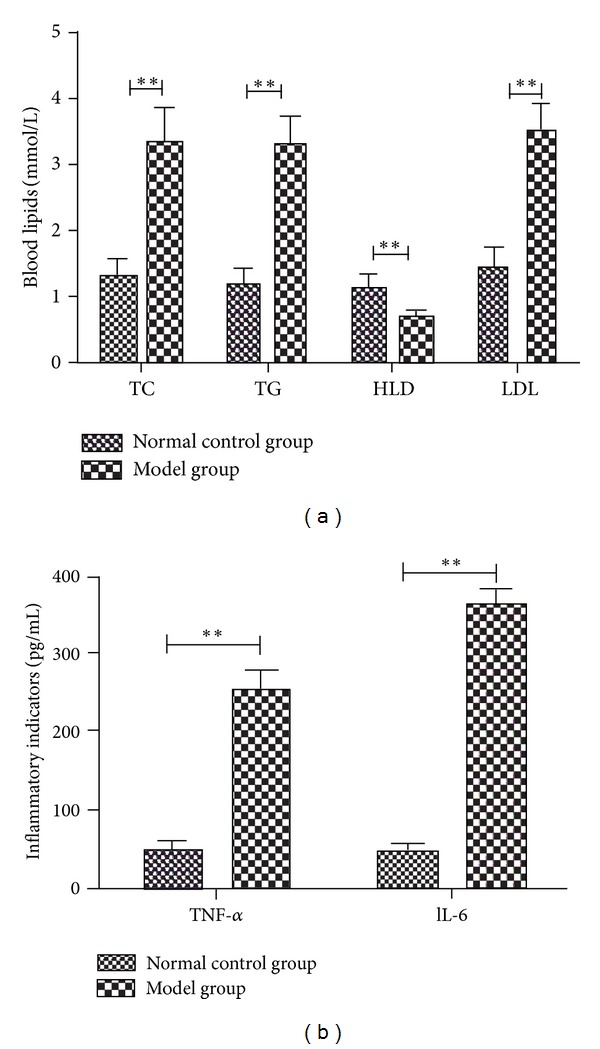
Blood biochemical variables in mice treated with LPS (i.v.) and atherogenic solution (gavage) for 8 weeks. **P < 0.01 versus normal control group.

After mice were treated with LPS (i.v.) and atherogenic solution (gavage) for 8 weeks, the histology and morphometric of thoracic aorta and myocardial tissues (HE staining) and the levels of TC, TG, HDL, LDL, TNF-*α*, and IL-6 in serum were analyzed. Compared with the nature control group, the result of HE staining showed that the aortic walls were thickened (Figure 3), and the myocardial cells became hypertrophic and arranged loosely (Figure 3). Additionally mononuclear infiltrate both in thoracic aorta and myocardial tissues in the model group were observed (Figure 3). The TC and TG serum levels of the model group were significantly higher than those of the normal control group (TC: 3.33 ± 0.54 versus 1.31 ± 0.24, TG: 3.28 ± 0.45 versus 1.16 ± 0.31, *P* < 0.01, for all) (Figure 4). High fat solution reduced HDL-C level (1.13 ± 0.25 versus 0.68 ± 0.13, *P* < 0.01) and increased LDL-C level (1.42 ± 0.34 versus 3.49 ± 0.41, *P* < 0.01). The expression levels of TNF-*α* and IL-6 were higher in the model group (TNF-*α*: 46.55 ± 8.35 versus 257.32 ± 18.95, IL-6: 47.47 ± 7.48 versus 360.07 ± 19.47, *P* < 0.01, for all) (Figure 4). 

**Figure 5:**
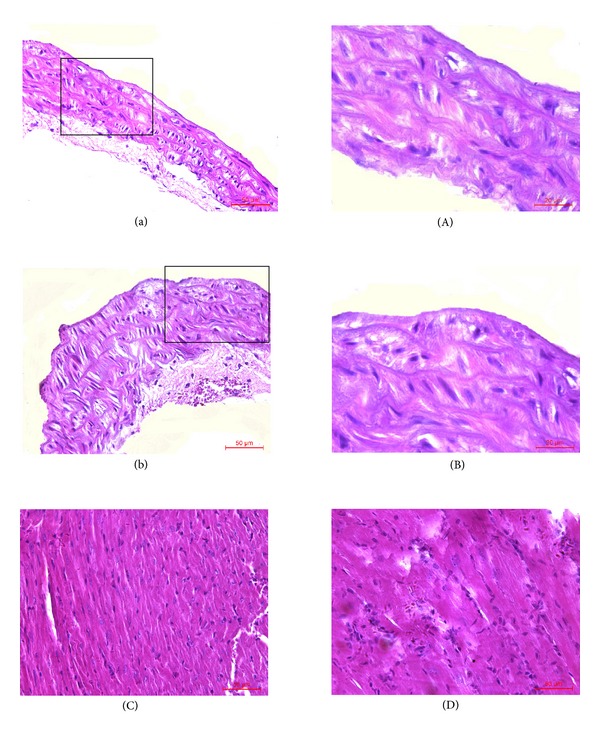
Histological and morphometric observation of thoracic aorta and myocardial tissues from atherosclerotic mice treated with anti-inflammatory effective parts of housefly larvae. (a) Thoracic aorta in anti-inflammatory effective parts of housefly larvae treatment group (×200). (b) Thoracic aorta in negative control group (×200). (A) Thoracic aorta in anti-inflammatory effective parts of housefly larvae treatment group (×400). (B) Thoracic aorta in negative control group (×400). (C) Myocardial tissues in anti-inflammatory effective parts of housefly larvae treatment group (×400). (D) Myocardial tissues in negative control group (×400).

**Figure 6:**
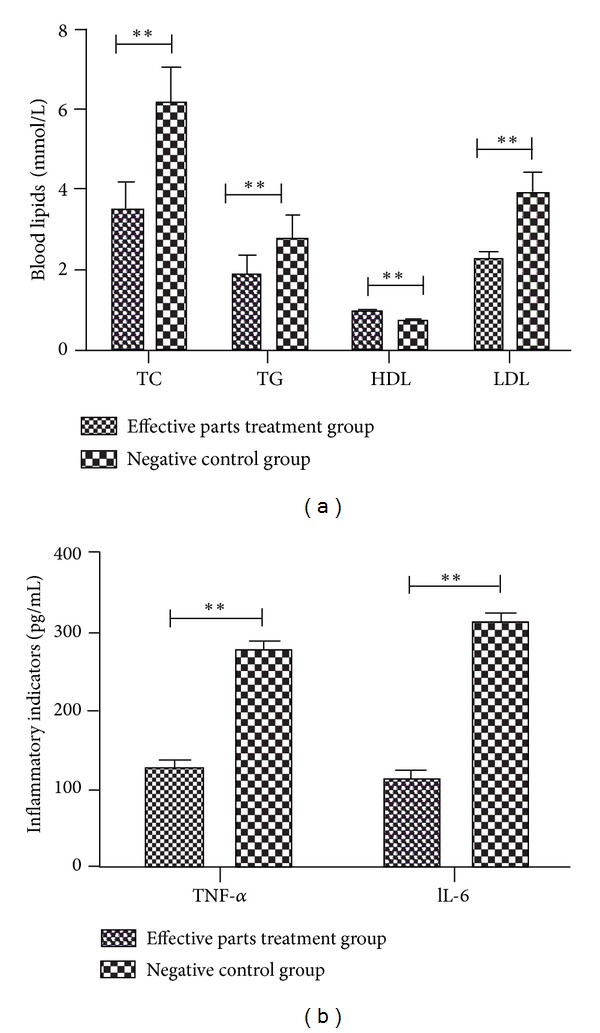
Blood biochemical variables in anti-inflammatory effective parts of housefly larvae treated group and negative control group. **P < 0.01 versus negative control group.

After treated with or without the anti-inflammatory effective parts for 4 weeks, compared with the negative control group, the size of atherosclerotic lesions in aortas was reduced and the inflammatory infiltrates both in thoracic aorta and myocardial tissues were decreased in effective parts treated group (Figure 5). And also except HDL which was increased (0.93 ± 0.04 versus 0.74 ± 0.03, *P* < 0.01), the other inflammatory and lipid parameters associated with atherosclerosis were reduced (TNF-*α*: 126.32 ± 11.08 versus 275.26 ± 12.35, IL-6: 114.66 ± 7.98 versus 314.33 ± 15.68, TC: 3.52 ± 0.68 versus 6.12 ± 1.04, TG: 1.88 ± 0.42 versus 2.79 ± 0.56, LDL: 2.23 ± 0.25 versus 3.87 ± 0.63, *P* < 0.01, for all) (Figure 6). 

### 3.4. Affect of Expression and Nuclear Transfer of NF-*κ*B p65 of Effective Parts

**Figure 7:**
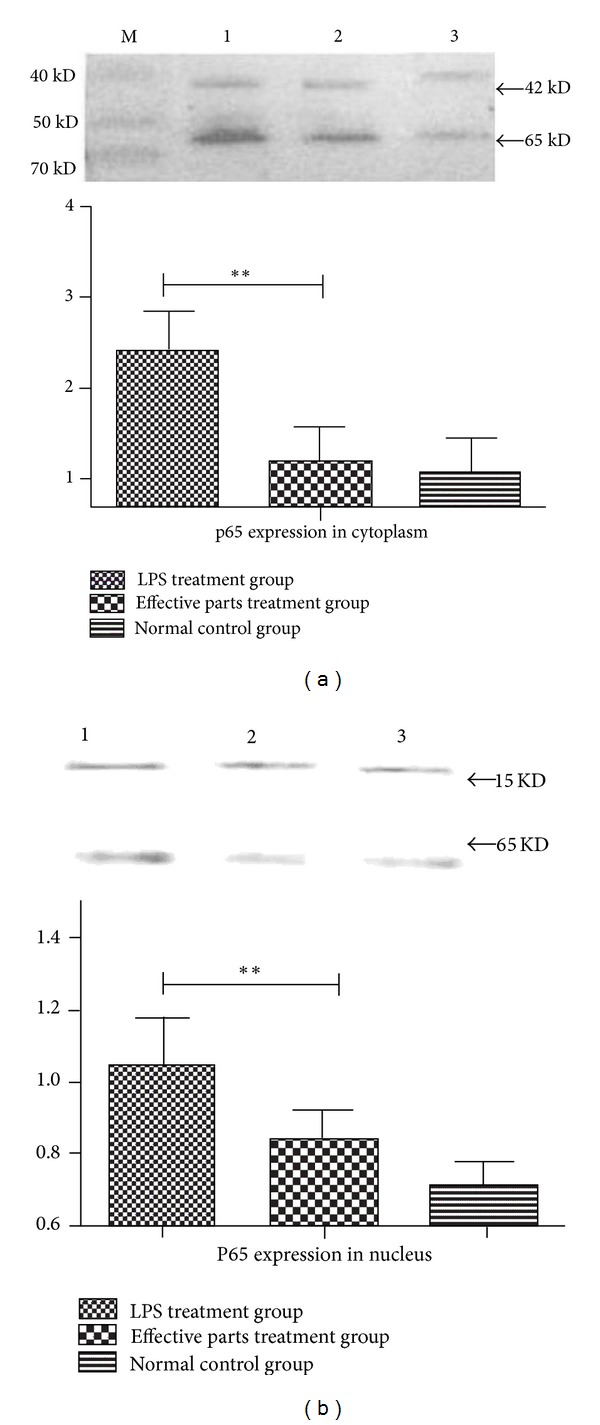
Effects of anti-inflammatory effective parts of housefly larvae on protein expression of NF-*κ*B p65 in LPS-induced RAW264.7 macrophages measured by Western blot. (a) Anti-inflammatory effective parts of housefly larvae decreased LPS-induced p65 expression in cytoplasm. *β*-actin was set as control normalization. Lane M: protein mark, lane 1: LPS-treated group, lane 2: anti-inflammatory effective parts of housefly larvae treated group, lane 3: natural control group. (b) Anti-inflammatory effective parts of housefly larvae inhibited LPS-induced p65 expression in nucleus. Histon H 3.1 was set as control normalization. Lane 1: LPS-treated group, lane 2: anti-inflammatory effective parts treatedgroup, lane 3: natural control group. **P < 0.05 versus LPS-treated group.

RAW264.7 macrophages were treated with or without effective parts and incubated for 18 h with LPS. The protein expressions of NF-*κ*B p65 in nucleus and cytoplasm were measured by western blot assay. It was demonstrated that LPS stimulation resulted in significant increase of p65 in nucleus and cytoplasm compared with that of natural control group (*P* < 0.01, Figure 7). Effective parts exerted potent inhibitory effect on expression of p65 in nucleus and cytoplasm compared with that in LPS-treated group (*P* < 0.01, Figure 7).

**Figure 8:**
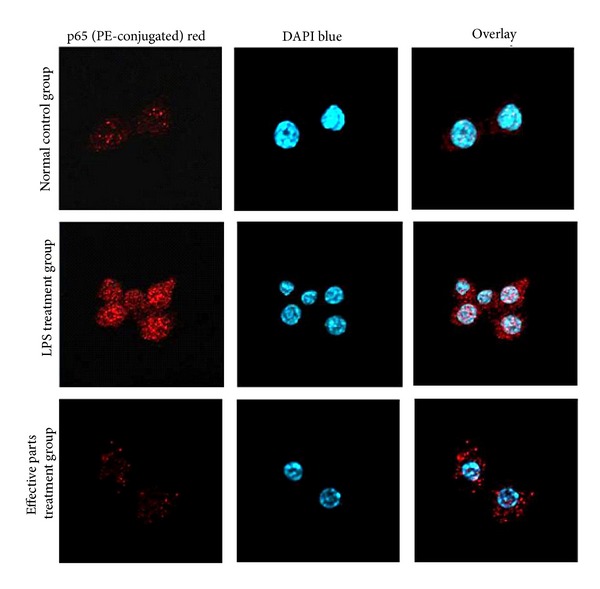
Immunofluorescent images of NF-*κ*B p65 under the laser scanning confocal microscopy in anti-inflammatory effective parts of housefly larvae treated group, negative control group, and normal control group. (1000x). NF-*κ*B p65 was stained with PE (red) and nuclei were stained with DAPI (blue).

To confirm that effective parts inhibit LPS-induced nuclear translocation of NF-*κ*B p65 protein in RAW264.7 macrophage, confocal microscopy was used to visualise the expression of p65 in cytoplasm and nucleus (Figure 8). The results also showed that the expressions of p65 both in cytoplasm and nucleus were reduced significantly (red fluorescence intensity declined, *P* < 0.01, data not show) compared to those in the LPS-treated group.

## 4. Discussion

Traditional medicines such as traditional Chinese medicine use a great array of insects and their products as drugs. Insects “are a kind of large, unexplored, and unexploited sources containing useful compounds for modern medicine” [10]. In china, housefly (*Musca domestica*) larvae have been used clinically to cure inflammation and inflammation-related diseases, such as osteomyelitis, decubital necrosis, lip boil, and ecthyma ever since Ming Dynasty (1368 A. D) [4]. During the past decade, due to the antibacterial activity of housefly larvae and its products, they have been used mainly in preservation [11], environmental protection [12], and inhibition of multiresistant bacteria [13]. In recent years, several reports suggested that atherosclerosis is a chronic inflammatory disease [14, 15] that can be accelerated by microbial infection in its early phase [16, 17]. Based on the above information, the antiatherosclerotic effect of the protein-enriched extracts of housefly larvae had been previously studied. On the basis of preliminary studies, the present study tested the effect of anti-inflammatory effective parts on atherosclerosis *in vivo*, and explored the anti-inflammatory mechanism* in vitro*.

In the present work, Gel-filtration chromatography (GFC) was used for proteins segregation in order of molecular size: large to small, which is similar to the method used by purification of a specific *β*-glucosidase from the digestive fluid of larvae of palm weevil (*Rhynchophorus palmarum*) [18], and small cationic protein from Tobacco Hornworm *(Manduca sexta)* [19]. Compared with other methods such as organic solvent precipitation [20], salting out precipitation [21], and high pressure/performance liquid chromatography (HPLC) [22], gel-filtration was the most promising method with the advantages of high specificity and efficiency. Gel filtration was carried out with fine grade Sephadex G-75 which had optimum range of fractionation (3,000–70,000). The crude protein-enriched extracts of housefly larvae were tested by SDS-page in previous studies which showed that there were three parts of proteins with similar weight of molecule which was consistent with the results of the present study. In the present study, three main parts of extracts were obtained based on the different molecular weights. So in the next step, the anti-inflammatory activity screening of all the three main parts of extracts was carried out *in vitro*.

The stimulation of macrophages induced by LPS is a useful protocol *in vitro *screening to identify new anti-inflammatory compounds [23]. The release of TNF-*α* and IL-6 could be used as indicators for anti-inflammatory activity [24]. In this study, LPS-stimulated RAW264.7 macrophages were used and the levels of TNF-*α* and IL-6 were investigated. The results of anti-inflammatory activity showed that the part III (small molecular weight) could reduce pseudopodia formation and the levels of TNF-*α* and IL-6 significantly. So this part was considered as the anti-inflammatory effective parts of housefly larvae.

With these *in vitro* results, the antiatherosclerotic properties in mouse of the anti-inflammatory effective parts of housefly larvae were explored. The atherosclerosis model was established by LPS (i.v.) and atherogenic solution (gavage) for 8 weeks. Although most mouse strains are highly resistant to atherosclerosis, when the atherosclerosis was induced by LPS and atherogenic diet for several weeks, the animal model could be established [25]. In the present study, the atherogenic diet was instead by atherogenic solution to guarantee that every animal could intake the same amount of cholesterol and fat and ensure the atherosclerotic model could be built quickly. Then the atherosclerotic mice were treated with the anti-inflammatory effective parts for 4 weeks by oral gavage. The hypothesis that the anti-inflammatory effective parts of housefly larvae had the effect of antiatherosclerosis *in vivo* was confirmed. The small molecular weight parts were initially postulated to be as antimicrobial peptides [26]. Although the present study was not aimed at optimizing the metabolic stability, approaches were available for the medicinal chemist to increase the metabolic resistance against degradation as described recently [27]. So the effect of anti-atherosclerosis *in vivo* of effective parts could be explained.

Today, atherosclerosis is recognized as a complex disease with serious inflammation. Consistent with its key role in coordinating inflammatory responses, numerous studies have suggested that the transcription factor nuclear factor *κ*B (NF-*κ*B) is one of the most important proinflammatory pathways in atherogenesis [28, 29]. It was reported that TNF-*α* expression was mainly regulated by the transcription factor NF-*κ*B [30]. In the present work, the level of TNF-*α* was decreased *in vitro *and *in vivo*, suggesting that regulation of NF-*κ*B signal pathway might be one of the possible mechanisms underlying anti-inflammatory effect of effective parts. In order to examine this possibility, effects on expression and nuclear transfer of NF-*κ*B p65 of anti-inflammatory effective parts of housefly larvae were detected. The results indicated that the expression and nuclear transfer of NF-*κ*B p65 were inhibited. In fact, entomic NF-*κ*B activation in response to immune challenge was homologous or related to molecules found in mammalian signal pathways activated during innate immune defence. Complex signal pathways regulate the innate immune system of insects, with NF-*κ*B transcription factors playing a key role in the activation of antimicrobial peptides and other immune genes [31]. 

In a further study, the composition and structure of the anti-inflammatory effective parts of housefly larvae will be explored by high-performance liquid chromatography tandem mass spectrometry (HPLC-MS/MS), sequencing, and bioinformatics analysis.

## 5. Conclusion

Anti-inflammatory effective parts of housefly larvae have the function of antiatherosclerosis in the mouse. There is a possibility that the mechanism could be associated with the inhibition of expression and nuclear transfer of NF-*κ*B p65.

## Figures and Tables

**Table 1:** Effect of different gel-filtration chromatography fractions of housefly larvae extracts on the secretion of TNF-*α* and IL-6 in LPS-stimulated RAW 264.7 cells. (pg/mL, *n* = 6).

	LPS−	LPS+	LPS+ part I	LPS+ part II	LPS+ part III
TNF-*α*	256.5 ± 8.7	1847.9 ± 27.2	1795.5 ± 27.1	1579.0 ± 32.2**	665.1 ± 12.9^∗∗##^
IL-6	82.8 ± 4.4	632.5 ± 8.2	624.7 ± 4.8	562.3 ± 11.6**	131.7 ± 5.9^∗∗##^

**P < 0.01 versus LPS group, ^##^ *P* < 0.01 versus part II group.
